# The MapCHECK Measurement Uncertainty function and its effect on planar dose pass rates

**DOI:** 10.1120/jacmp.v17i2.5995

**Published:** 2016-03-08

**Authors:** Daniel W. Bailey, Jason D. Spaans, Lalith K. Kumaraswamy, Matthew B. Podgorsak

**Affiliations:** ^1^ Department of Radiation Oncology Northside Hospital Cancer Institute Atlanta GA USA; ^2^ Department of Radiation Medicine Roswell Park Cancer Institute Buffalo NY USA; ^3^ Department of Physiology and Biophysics State University of New York at Buffalo Buffalo NY USA

**Keywords:** radiation therapy, IMRT QA, quality assurance, dosimetry, dose verification, diode array, planar dose

## Abstract

Our study aimed to quantify the effect of the Measurement Uncertainty function on planar dosimetry pass rates, as measured and analyzed with the Sun Nuclear Corporation MapCHECK 2 array and its associated software. This optional function is toggled in the program preferences of the software (though turned on by default upon installation), and automatically increases the dose difference tolerance defined by the user for each planar dose comparison. Dose planes from 109 static‐gantry IMRT fields and 40 VMAT arcs, of varying modulation complexity, were measured at 5 cm water‐equivalent depth in the MapCHECK 2 diode array, and respective calculated dose planes were exported from a commercial treatment planning system. Planar dose comparison pass rates were calculated within the Sun Nuclear Corporation analytic software using a number of calculation parameters, including Measurement Uncertainty on and off. By varying the percent difference (%Diff) criterion for similar analyses performed with Measurement Uncertainty turned off, an effective %Diff criterion was defined for each field/arc corresponding to the pass rate achieved with Measurement Uncertainty turned on. On average, the Measurement Uncertainty function increases the user‐defined %Diff criterion by 0.8%‐1.1% for 3%/3 mm analysis, depending on plan type and calculation technique (corresponding to an average change in pass rate of 1.0%‐3.5%, and a maximum change of 8.7%). At the 2%/2 mm level, the Measurement Uncertainty function increases the user‐defined %Diff criterion by 0.7%‐1.2% on average, again depending on plan type and calculation technique (corresponding to an average change in pass rate of 3.5%‐8.1%, and a maximum change of 14.2%). The largest increases in pass rate due to the Measurement Uncertainty function are generally seen with poorly matched planar dose comparisons, while the function has a notably smaller effect as pass rates approach 100%. The Measurement Uncertainty function, then, may substantially increase the pass rates for planar dose comparisons. Meanwhile, the types of uncertainties incorporated into the function (and their associated quantitative estimates, as described in the software user's manual) may not be an accurate estimation of actual measurement uncertainty, depending on the user's measurement conditions. Pass rates listed in published reports, comparisons between institutions or simply separate workstations, or comparisons with the calculation methods of other vendors, should clearly indicate whether or not the Measurement Uncertainty function is used, since it has the potential to substantially inflate pass rates for typical IMRT and VMAT dose planes.

PACS number(s): 87.55.Qr, 87.56.Fc

## I. INTRODUCTION

Planar diode arrays are a standard tool for myriad external beam quality assurance tests, most popularly for patient‐specific dosimetric verification of intensity‐modulated radiation therapy fields,^1^, ^2^ for both static^3^, ^4^ and rotational^5^, ^6^ delivery techniques. One of the diode arrays most commonly used for planar dose measurement is the MapCHECK^7^, ^8^ device (and its upgrade, the MapCHECK 2)^9^, ^10^, ^11^ by Sun Nuclear Corporation of Melbourne, FL, accompanied by its analytical software component (i.e., “MapCHECK” up to version 5.2; “SNC Patient” thereafter). The MapCHECK and SNC Patient softwares allow the user to quantitatively compare measured and calculated dose planes, utilizing the gamma analysis and distance‐to‐agreement (DTA) techniques described elsewhere in the literature.^12^, ^13^, ^14^

In a previous report, it was demonstrated that, rather than describing such a dosimetric comparison with a single pass rate, the comparison of any two respective measured and calculated planar dose distributions is actually characterized by a spectrum of pass rates dependent on parameters such as calculation method, percent difference (%Diff) and DTA tolerances, choice of percent difference normalization value, detector spatial resolution and individual diode position with respect to the beam's eye view, absolute or relative dose comparison, and the user's definition of region of interest and/or choice of applied dose threshold (i.e., dose level below which individual diode responses are not incorporated into the pass rate).^(14)^ For this reason, pass rates should be reported (especially between institutions, e.g., in publications) with a comprehensive description of exactly how said pass rates are calculated.

An additional calculation parameter within the MapCHECK and SNC Patient software, which is often applied but seldom reported in the literature, is the “Measurement Uncertainty” function, which is turned on by default upon software installation and can be toggled in program preferences.^(15)^ The software manual describes this function as “a supplement to the user‐defined acceptance criteria … added to the percentage acceptance criterion defined by the user” to account for “various uncertainties in measurement.”^(15)^ In other words, this optional function automatically increases the dose difference tolerance selected by the user for DTA or gamma comparison of planar dose distributions, in an attempt to compensate for various sources of presumed uncertainty in MapCHECK or ArcCHECK array measurements. Because the Measurement Uncertainty value is added to the %Diff criterion chosen for gamma or DTA analysis, this function always increases the resultant pass rate. Task Group 119 of the American Association of Physicists in Medicine utilized the Measurement Uncertainty function in the analysis of their cross‐institutional data, stating that they “applied measurement uncertainty (a presumed measurement error of about 1% is included in the analysis, so that a nominal 3% dose difference can be 4%).”^(16)^

In the absence of quantitative data demonstrating the effect of the Measurement Uncertainty function on absolute dose pass rates, we determined to study a number of static‐gantry IMRT and RapidArc fields at various %Diff/DTA criteria levels and with/without Measurement Uncertainty to evaluate this parameter that is unique to the Sun Nuclear Corporation software.

## II. MATERIALS AND METHODS

The following treatment plans were used in this study, calculated with the Eclipse treatment planning system (TPS; AAA 10, Varian Medical Systems, Palo Alto, CA): five static‐gantry IMRT plans for head/neck (49 fields), nine static‐gantry IMRT plans for prostate (60 fields), ten RapidArc plans for head/neck (20 arcs), and ten RapidArc plans for prostate (20 arcs). Each field or arc was delivered on a Varian Trilogy linear accelerator system, and planar dose data were measured via MapCHECK 2 placed inside the MapPHAN^9^, ^10^, ^11^ water‐equivalent phantom with source‐to‐detector distance of 100 cm and water‐equivalent depth of 5 cm. Respective in‐phantom verification plans were created for each field or arc within the TPS and cross‐sectional dose planes (at 5 cm water‐equivalent depth) were exported in DICOM format for analysis with the SNC Patient software. For large IMRT fields requiring split carriages, each carriage was measured separately with the diode array, then merged and analyzed as one treatment field.

For brevity and clarity, we will refer to MapCHECK Measurement Uncertainty on and off as ${\text{MCU}}_{\text{on}}$ and ${\text{MCU}}_{\text{off}}$, respectively. Quantitative comparison of measured versus calculated dose planes was conducted within the SNC Patient software (version 6.2.0), first with the DTA method and then with the gamma method, using absolute dose mode, global percent difference normalization to the maximum planned point dose, and a 10% dose threshold (effectively limiting the analysis to within the collimator jaws). The “Calculate Shift” function was not used, so no geometric optimization of pass rate was employed. In addition, pass rates (i.e., percentage of measured points with gamma value less than unity) were recorded with DTA criterion of 2 mm while the %Diff criterion was varied from 1% to 4% in half‐percent increments, and again with DTA criterion of 3 mm with the same variation in %Diff. To quantify the effect of Measurement Uncertainty, pass rates with each selection of %Diff/DTA criterion were calculated both with ${\text{MCU}}_{\text{on}}$ and ${\text{MCU}}_{\text{off}}$. These data were used to approximate an effective percent difference $\left(\%{\text{Diff}}_{\text{eff}}\right)$ for each pass rate calculated with ${\text{MCU}}_{\text{on}}$: for a given DTA criterion and ${\text{MCU}}_{\text{off}}$, the pass rates for varying %Diff were linearly interpolated, and the %Diff value corresponding to the pass rate calculated with ${\text{MCU}}_{\text{on}}$ was defined as the effective percent difference criterion for that comparison.

## III. RESULTS AND DISCUSSION

Tables 1 and 2 summarize the results for prostate and head/neck static‐gantry IMRT using DTA and gamma analysis, respectively, both with 3%/3 mm criteria. Tables 3 and 4 present the same analysis but at the 2%/2 mm level. Similarly, Tables 5 through 8 present the results for RapidArc plans. These tables all indicate similar results: at the 3%/3 mm analysis level, the Measurement Uncertainty function increases user‐defined %Diff criterion to an effective %Diff between 3.8% to 4.1% on average, depending on plan type and calculation technique. Meanwhile, at the 2%/2 mm analysis level, the Measurement Uncertainty function yields an effective %Diff of approximately 2.7%‐3.2% on average. In other words, the 3%/3 mm pass rate with ${\text{MCU}}_{\text{on}}$ is approximately equivalent to a 4%/3 mm pass rate calculated with ${\text{MCU}}_{\text{off}}$. For individual fields, the effective %Diff can be as high as 6% when analyzing fields at the 3%/3 mm level, and as high as 5.8% when using the 2%/2 mm criteria. The maximum observed change in pass rate for static‐gantry IMRT fields due to the Measurement Uncertainty function was +8.7% at 3%/3 mm and +14.2% at the 2%/2 mm level. For RapidArc plans, the maximum observed change in pass rate due to the Measurement Uncertainty function was +8.7% at 3%/3 mm and +13.1% at the 2%/2 mm level.

**Table 1:** MapCHECK Uncertainty results for IMRT with DTA 3%/3 mm

*Statistics*	*3%/3 mm* ${\text{MCU}}_{\text{on}}$	*3%/3 mm* ${\text{MCU}}_{\text{off}}$	*3%/3 mm (on ‐ off)*	$\%{\text{Diff}}_{\text{eff}}$ *for 3%/3 mm*
Prostate	average	95.5	92.3	3.3	4.1
	minimum	84.4	77.1	0.0	3.0
	maximum	100.0	99.5	8.7	6.0
	SD	4.0	5.8	2.2	0.5
H&N	average	96.1	94.2	1.9	4.1
	minimum	90.8	85.9	0.0	3.2
	maximum	99.3	98.7	5.3	6.0
	SD	2.1	2.7	1.1	0.5

a SD=standard deviation

**Table 2:** MapCHECK Uncertainty results for IMRT with gamma 3%/3 mm

*Statistics*	*3%/3 mm* ${\text{MCU}}_{\text{on}}$	*3%/3 mm* ${\text{MCU}}_{\text{off}}$	*3%/3 mm (on ‐ off)*	$\%{\text{Diff}}_{\text{eff}}$ *for 3%/3 mm*
Prostate	average	97.2	95.5	1.7	3.8
	minimum	89.8	83.8	0.0	3.1
	maximum	100.0	100.0	7.8	4.0
	SD	2.8	4.1	1.8	0.3
H&N	average	97.4	96.4	1.0	3.8
	minimum	92.6	90.3	0.0	3.0
	maximum	100.0	99.7	4.0	6.1
	SD	1.7	2.3	1.0	0.5

SD=standard deviation

**Table 3:** MapCHECK Uncertainty results for IMRT with DTA 2%/2 mm

*Statistics*	*2%/2 mm* ${\text{MCU}}_{\text{on}}$	*2%/2 mm* ${\text{MCU}}_{\text{off}}$	*2%/2 mm (on ‐ off)*	$\%{\text{Diff}}_{\text{eff}}$ *for 2 %/2 mm*
Prostate	average	84.9	76.8	8.1	3.2
	minimum	64.4	53.6	2.2	2.4
	maximum	98.7	92.7	14.2	5.8
	SD	9.8	11.0	3.1	0.5
H&N	average	89.5	84.4	5.0	3.0
	minimum	81.0	73.3	1.9	2.4
	maximum	95.8	92.6	8.7	5.6
	SD	3.3	4.5	1.8	0.5

SD=standard deviation

**Table 4:** MapCHECK Uncertainty results for IMRT with gamma 2%/2 mm

*Statistics*	*2%/2 mm* ${\text{MCU}}_{\text{on}}$	*2%/2 mm* ${\text{MCU}}_{\text{off}}$	*2%/2 mm (on ‐ off)*	$\%{\text{Diff}}_{\text{eff}}$ *for 2 %/2 mm*
Prostate	average	87.1	81.6	5.5	2.8
	minimum	69.9	60.7	0.7	2.3
	maximum	99.4	96.8	11.8	3.0
	SD	8.5	10.1	2.7	0.2
H&N	average	91.3	87.8	3.5	2.7
	minimum	83.0	77.7	1.2	2.3
	maximum	97.4	95.1	8.3	3.2
	SD	3.1	4.0	1.7	0.2

SD=standard deviation

**Table 5:** MapCHECK Uncertainty results for VMAT with DTA 3%/3 mm

*Statistics*	*3%/3 mm* ${\text{MCU}}_{\text{on}}$	*3%/3 mm* ${\text{MCU}}_{\text{off}}$	*3%/3 mm (on ‐ off)*	$\%{\text{Diff}}_{\text{eff}}$ *for 3 %/3 mm*
Prostate	average	96.2	92.8	3.4	3.8
	minimum	92.7	87.3	1.6	3.6
	maximum	98.7	96.5	5.5	4.1
	SD	2.0	2.7	1.1	0.2
H&N	average	95.8	92.2	3.5	3.8
	minimum	90.4	86.2	1.3	3.5
	maximum	99.5	97.7	8.7	4.1
	SD	2.4	3.4	1.7	0.1

SD=standard deviation

**Table 6:** MapCHECK Uncertainty results for VMAT with gamma 3%/3 mm

*Statistics*	*3%/3 mm* ${\text{MCU}}_{\text{on}}$	*3%/3 mm* ${\text{MCU}}_{\text{off}}$	*3%/3 mm (on ‐ off)*	$\%{\text{Diff}}_{\text{eff}}$ *for 3 %/3 mm*
Prostate	average	97.8	95.7	2.2	3.8
	minimum	94.6	89.7	0.8	3.3
	maximum	100.0	98.6	4.9	4.0
	SD	1.7	2.2	0.9	0.2
H&N	average	97.4	95.1	2.3	3.8
	minimum	92.9	90.8	0.7	3.4
	maximum	100.0	99.2	5.1	4.0
	SD	1.7	2.5	1.1	0.2

SD=standard deviation

**Table 7:** MapCHECK Uncertainty results for VMAT with DTA 2%/2 mm

*Statistics*	*2%/2 mm* ${\text{MCU}}_{\text{on}}$	*2%/2 mm* ${\text{MCU}}_{\text{off}}$	*2%/2 mm (on ‐ off)*	$\%{\text{Diff}}_{\text{eff}}$ *for 2 %/2 mm*
Prostate	average	87.8	81.2	6.5	2.7
	minimum	78.4	73.0	2.6	2.4
	maximum	92.1	86.5	9.4	3.2
	SD	3.5	4.1	1.8	0.2
H&N	average	87.5	79.8	7.7	2.8
	minimum	80.3	69.8	2.6	2.6
	maximum	94.1	91.5	13.1	3.0
	SD	4.2	6.1	2.6	0.1

SD=standard deviation

**Table 8:** MapCHECK Uncertainty results for VMAT with gamma 2%/2 mm

*Statistics*	*2%/2 mm* ${\text{MCU}}_{\text{on}}$	*2%/2 mm* ${\text{MCU}}_{\text{off}}$	*2%/2 mm (on ‐ off)*	$\%{\text{Diff}}_{\text{eff}}$ *for 2 %/2 mm*
Prostate	average	90.6	85.2	5.3	2.7
	minimum	83.8	77.9	2.8	2.4
	maximum	95.2	91.8	9.3	2.9
	SD	3.5	4.3	1.5	0.1
H&N	average	90.2	84.5	5.8	2.7
	minimum	83.1	73.4	2.6	2.5
	maximum	96.4	93.8	10.3	2.9
	SD	4.2	5.5	1.9	0.1

SD=standard deviation

**Figure 1:**
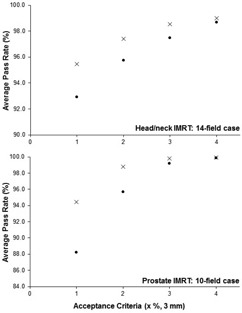
Static‐gantry IMRT planar dose comparisons for a head/neck (top) and prostate (bottom) case. Absolute dose gamma evaluations performed with variable percent difference criterion as indicated on the horizontal axes, and fixed distance‐to‐agreement criterion of 3 mm. Average pass rates calculated with Measurement Uncertainty on are indicated by “x” markers, while similar averages calculated with Measurement Uncertainty off are indicated by “dot” markers.

**Figure 2:**
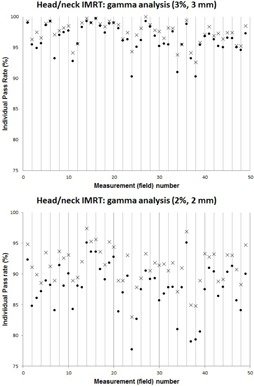
Static‐gantry IMRT planar dose comparisons for all 49 head/neck fields tested in this report, first with gamma analysis of 3%/3 mm (top) and secondly with 2%/2 mm criteria (bottom). Each vertical column represents a single planar dose comparison of a measured field vs. its respective dose plane calculated in the TPS, resulting in one pass rate with ${\text{MCU}}_{\text{on}}$ (x) and the other with ${\text{MCU}}_{\text{off}}$ (dot).

Figures 1 and 2allow visualization of the spread of pass rates for different gamma criterion (namely, 3%/3 mm and 2%/2 mm), as well as the effect of toggling the Measurement Uncertainty function. In each of the figures, the “x” markers indicate data collected with the ${\text{MCU}}_{\text{on}}$ while the “dot” markers indicate data collected with ${\text{MCU}}_{\text{off}}$. Figure 1 (top) shows the average pass rates for a static‐gantry IMRT head/neck case (14 fields total) and Fig. 1 (bottom) shows the average pass rates for a static‐gantry IMRT prostate case (10 fields total). For these calculations, the percent difference criterion was varied from 1% to 4%, as indicated on the horizontal axes, while the distance‐to‐agreement criterion was fixed at 3 mm. Meanwhile, Fig. 2 displays the individual pass rates for all 49 static‐gantry IMRT head/neck fields tested for this report, first with gamma analysis of 3%/3 mm (top) and secondly with 2%/2 mm criteria (bottom). Each vertical column displays a pair of pass rates for an individual planar dose comparison (measured vs. TPS), one with ${\text{MCU}}_{\text{on}}$ (x) and the other with ${\text{MCU}}_{\text{off}}$ (dot) (i.e., the only change is made by toggling the Measurement Uncertainty function). From these figures, two general trends are noteworthy and complimentary to the tabular data discussed above: the Measurement Uncertainty function may result in large increases in pass rate for poorly matching planar dose comparisons; however, the Measurement Uncertainty function has a smaller effect on already high pass rates, evidently due to the asymptotic behavior of the gamma calculation when approaching 100%.

At the time of final submission of this manuscript, the most current versions of the SNC Patient software (beginning with version 6.2.3, released after the data in this work were collected) include an updated Measurement Uncertainty function, as detailed identically in the current versions of the Sun Nuclear Corporation help manuals (either “MapCHECK Help,” “MapCHECK 2 Help,” or “ArcCHECK Help,” depending on the user's device). While the basic functionality of the Measurement Uncertainty feature remains consistent with the discussion in this work, a constant value was assumed for the magnitude of the correction applied directly to (i.e., increasing) the user‐selected dose difference percentage: 0.8% for absolute dose comparison and 0.5% for relative dose comparison with MapCHECK and MapCHECK 2. According to these vendor manuals, the Measurement Uncertainty evidently behaves similarly when applied to ArcCHECK measurements, with slightly higher constant values of 1.0% for absolute dose comparison and 0.7% for relative dose comparison, added directly to the user‐defined dose difference comparison criterion. Since the Measurement Uncertainty function in previous versions of the software was allowed to take on variable values depending on each measured data point, the magnitude of the function in the latest version of the software, or in any future update to the software, may potentially be different.

Users of the MapCHECK and SNC Patient software tools should be aware that the Measurement Uncertainty function can result in substantially inflated pass rates for planar dose comparisons. While each institution included in the TG‐119 data pool employed Measurement Uncertainty for the sake of consistency (and not as an endorsement), it should be noted that this function adds another level of difficulty in comparing the pass rates calculated by the SNC software to pass rates calculated by other software analysis tools (e.g., from other vendors), or pass rates calculated by other institutions (or even other workstations within the same institution) that do not have the SNC software set up to employ the Measurement Uncertainty function. At the same time, several recent reports^17^, ^18^, ^19^, ^20^ indicate that inflated pass rates may actually mask errors in treatment planning and delivery, and this information should be weighed in the decision to use the Measurement Uncertainty function.

A typical indication of measurement uncertainty would tend to provide an interval of confidence in the given measurement. Namely, the actual uncertainty in any measured dose plane comparison indicates a pass rate potentially higher or lower than the value calculated without accounting for such uncertainties. However, because of the definition of the Measurement Uncertainty function, as an addition to the user‐defined %Diff tolerance level, it can only increase the calculated pass rate.^(20)^ Specifically, due to this application of estimated uncertainty, a measured dose point outside the user‐defined tolerance might be included as a passing point, while a dose point inside the tolerance criteria but with uncertainty enough to potentially be a failing point is never actually counted as a failing point. The SNC user's manual lists several uncertainties approximately quantified and included in the Measurement Uncertainty function. These include an array calibration correction (approximated from a study dealing with the original MapCHECK device),^(21)^ a correction for assumed accelerator output and room temperature differences between the time of array calibration and actual measurement, and a correction for an assumed error in setup source‐to‐surface distance (SSD). The user should decide whether or not these enumerated uncertainties (and their approximate values used to supplement the %Diff criteria) are applicable to one's own measurement conditions. For example, a default 2 mm SSD correction may not be appropriate for a carefully set up device coupled to a quality‐controlled accelerator, while an automatic pass rate adjustment to account for assumed output fluctuation and assumed temperature difference between the time of measurement and the time of calibration may not be accurate for a diode array that is frequently calibrated. If the actual use of the diode array does not match the assumed corrections incorporated into the Measurement Uncertainty function, then the use of the function does not enhance the accuracy of subsequent calculated pass rates.

From the current literature on the subject, planar dose QA pass rates are quite variable even for a single treatment field, and are dependent on device used for measurement, resolution of detector and detector position (i.e., sampling of measured points within the field), region of interest, and dose/DTA tolerance criteria, along with all of the physical uncertainties that accompany these types of measurements. However, this work demonstrates that even identical measurements may yield significantly different pass rates depending on the specifics of the vendor's algorithm utilized to implement the gamma evaluation. It would be useful to all users if each vendor would provide an analysis of expected returned pass rate values based upon a universally employed dataset, much like the dataset provided alongside the TG‐119 report, giving the user an indication of any pass rate inflation or deflation inherent to the vendor's calculation algorithm.

## V. CONCLUSION

This study demonstrates that the Measurement Uncertainty function within the SNC planar dose comparison software may substantially inflate the pass rates for planar dose comparisons, by as much as 9%‐14% depending on plan type and calculation technique. Since this function is unique to the MapCHECK and SNC Patient software tools, use of the Measurement Uncertainty function should be clearly indicated, especially in published reports. Meanwhile, the types of uncertainties incorporated into the function (and their associated quantitative estimates, as described in the software user's manual) may not be an accurate estimation of actual measurement uncertainty, depending on the user's measurement conditions. Consequently, each user should carefully consider whether or not the application of the Measurement Uncertainty function matches his or her own measurement techniques and conditions.

## COPYRIGHT

This work is licensed under a Creative Commons Attribution 4.0 International License.
